# Proteomic Analysis on Anti-Proliferative and Apoptosis Effects of Curcumin Analog, 1,5-bis(4-Hydroxy-3-Methyoxyphenyl)-1,4-Pentadiene-3-One-Treated Human Glioblastoma and Neuroblastoma Cells

**DOI:** 10.3389/fmolb.2021.645856

**Published:** 2021-04-30

**Authors:** Yee Qian Lee, Pathmanathan Rajadurai, Faridah Abas, Iekhsan Othman, Rakesh Naidu

**Affiliations:** ^1^Jeffrey Cheah School of Medicine and Health Sciences, Monash University Malaysia, Subang Jaya, Malaysia; ^2^Laboratory of Natural Products, Faculty of Science, University Putra Malaysia, Seri Kembangan, Malaysia; ^3^Department of Food Science, Faculty of Food Science and Technology, University Putra Malaysia, Seri Kembangan, Malaysia

**Keywords:** glioblastoma, neuroblastoma, diarylpentanoids, cytotoxicity, anti-proliferation, apoptosis, shotgun proteomics

## Abstract

Curcumin analogs with excellent biological properties have been synthesized to address and overcome the poor pharmacokinetic profiles of curcumin. This study aims to investigate the cytotoxicity, anti-proliferative, and apoptosis-inducing ability of curcumin analog, MS13 on human glioblastoma U-87 MG, and neuroblastoma SH-SY5Y cells, and to examine the global proteome changes in these cells following treatment. Our current findings showed that MS13 induced potent cytotoxicity and anti-proliferative effects on both cells. Increased caspase-3 activity and decreased bcl-2 concentration upon treatment indicate that MS13 induces apoptosis in these cells in a dose- and time-dependent manner. The label-free shotgun proteomic analysis has defined the protein profiles in both glioblastoma and neuroblastoma cells, whereby a total of nine common DEPs, inclusive of glyceraldehyde 3-phosphate dehydrogenase (GAPDH), alpha-enolase (ENO1), heat shock protein HSP 90-alpha (HSP90AA1), Heat shock protein HSP 90-beta (HSP90AB1), Eukaryotic translation initiation factor 5A-1 (EFI5A), heterogenous nuclear ribonucleoprotein K (HNRNPK), tubulin beta chain (TUBB), histone H2AX (H2AFX), and Protein SET were identified. Pathway analysis further elucidated that MS13 may induce its anti-tumor effects in both cells via the common enriched pathways, “Glycolysis” and “Post-translational protein modification.” Conclusively, MS13 demonstrates an anti-cancer effect that may indicate its potential use in the management of brain malignancies.

## Introduction

Glioblastoma and neuroblastoma are the two most common neural and brain tumors among adults and children, respectively (de Weille, 2014). Glioblastoma, which is classified as Grade IV malignant gliomas by the World Health Organization (WHO) (Louis et al., 2007; Gupta and Dwivedi, 2017), has been well documented for its deadly mortality rate, with a 5-year survival rate of less than 50% (Delgado-López and Corrales-García, 2016). On the other hand, a neuroblastoma that originates from neural crest cells is well known for its heterogenous clinical outcomes, ranging from spontaneous regression to rapid metastasis with poor prognosis (Tomolonis et al., 2018). Multimodality treatment inclusive of surgery in combination with chemotherapy and radiotherapy remains as the most established therapeutic regime for both glioblastoma and neuroblastoma. Nonetheless, this therapeutic regime has yet to substantially improve the morbidity and mortality and, therefore, highlights the need for more powerful anti-cancer drugs.

Curcumin or diferuloylmethane is a hydrophobic polyphenol extracted from the rhizome of *Curcuma longa*. It is regarded as a “Generally Recognized as Safe” phytocompound by the Food and Drug Administrative (FDA) with good tolerability and safety profiles. Therefore, it is widely consumed as a spice, preservative, and coloring agent in Asian countries. Additionally, it also serves as an Ayurvedic medicine for ailments such as biliary and hepatic disorders, cough, abdominal pain, and rheumatism (Chattopadhyay et al., 2004). Curcumin facilitates a wide spectrum of biological activities, including anti-inflammatory, anti-oxidant, anti-parasitic, chemo-preventive, pro-apoptotic, and anti-bacterial properties through the modulation of a plethora of signaling molecules (Chattopadhyay et al., 2004). In 1987, the cytotoxic activity of curcumin was first demonstrated on leukemic lymphocytes by Kuttan et al. (1985). Since then, increasing interest was drawn to delineate the anti-cancer effects of curcumin on other types of cancers (Choudhuri et al., 2002; Jaiswal et al., 2002; Lin et al., 2007). Noteworthy, the cytotoxic and anti-proliferative effects of curcumin on glioblastoma cells (Karmakar et al., 2007) or its xenograft models (Perry et al., 2010) are important findings, suggesting possible therapeutic intervention approaches. Studies revealed that curcumin induced its cytotoxic potential by arresting the glioblastoma cells in the G2/M phase and, in turn, increased the sensitivity of cells toward radiation (Zanotto-Filho et al., 2012; Yu et al., 2015). Additionally, apoptotic (Huang et al., 2010) and cell signaling molecules (Yu et al., 2015) were also identified as the molecular targets for curcumin in glioblastoma cells. These encouraging evidences indeed showcased the therapeutic potential of curcumin in treating glioblastoma. However, limitations such as low bioavailability, rapid degradation, and short half-life have impeded the development of curcumin as a clinical drug. In this regard, numerous strategies, including the chemical structure modification, have been envisaged by researchers to overcome the poor pharmacokinetic profiles of curcumin.

Diarylpentanoids are products of chemical modification around the α,β-unsaturated diketone group in curcumin. These curcumin analogs that bear a five-carbon spacer in between the terminal aryl rings demonstrate better pharmacokinetic profiles and bioactivities. Several studies suggested that diarylpentanoid may be a better therapeutic option over its parent compound in treating cancers. For instance, GO-Y016, GO-Y030, and GO-Y031 demonstrated 30 times more potent growth-suppressive activity compared with curcumin, 5-fluorouracil, cisplatin, and irinotecan when treated on colorectal cancer cells (Ohori et al., 2006). Another selected diarylpentanoid (MS13) used in our present study that bears a five-carbon spacer with α,β-unsaturated ketone group between the terminal aryl rings also showed consistent findings, in which up to five times lower EC_50_ was revealed when treated on prostate (Citalingam et al., 2015) and cervical (Paulraj et al., 2015) cancer cells. Moreover, this compound was also found to induce cancer cell death by activating the apoptosis pathway in gastric (Yoshida et al., 2018) and colorectal (Cen et al., 2009) cancer cells. To date, no studies have reported on the anti-cancer activities and the mode of action of MS13 in human glioblastoma and neuroblastoma cells.

Hence, this study aims to investigate the cytotoxicity, anti-proliferative, and apoptosis effects of MS13 against human glioblastoma and neuroblastoma cells. We extended our study in analyzing the differentially expressed proteins (DEPs) in human glioblastoma and neuroblastoma cells upon exposure to MS13 relative to untreated cells. The high-throughput label-free shotgun proteomic analysis revealed several commonly DEPs in both glioblastoma and neuroblastoma cells, in which these data further provided an insight into the plausible pathways modulated by MS13 as a potential anti-cancer agent in treating brain malignancies in the future. This study, we believe, is the first to investigate the anti-cancer effects of MS13 on human glioblastoma and neuroblastoma cells from the proteomic perspective.

## Materials and Methods

### Cell Culture and Maintenance

Human glioblastoma (U-87 MG) cell line was purchased from ElabScience^®^ (Wuhan, China), whereas human neuroblastoma (SH-SY5Y) cell line and normal human epithelial hepatocytes, WRL-68 were obtained from the American Type Culture Collection (ATCC, Rockville, MD, United States). The cell lines were grown in Eagle’s Minimum Essential Medium (EMEM, Corning, Mediatech, Manassas, VA, United States) supplemented with 10% fetal bovine serum (FBS, Gibco^®^, NY, United States) and 1% antibiotic penicillin (100 U/ml)/streptomycin (100 μg/ml) (Gibco^®^, NY, United States). The cells were maintained at 37°C in a humidified incubator with 5% CO_2_.

### Preparation of Curcumin Analog (MS13) and Curcumin

**FIGURE 1:**
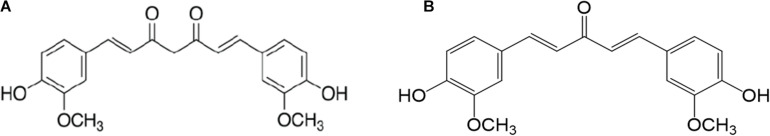
The chemical structure of **(A)** curcumin and **(B)** 1,5-bis(4-hydroxy-3-methoxyphenyl)-1,4-pentadiene-3-one (MS13).

MS13 or 1,5-bis(4-hydroxy-3-methoxyphenyl)-1,4-pentadiene-3-one was synthesized by coupling ketone with aldehyde in the ratio of 1:2, via base-catalyzed aldol condensation (Hosoya et al., 2012). Curcumin was purchased from commercial sources (Sigma Aldrich, St. Louis, MA, United States). Both MS13 and curcumin were dissolved in dimethyl sulfoxide (DMSO, Sigma Aldrich, St. Louis, MA, United States) into 50 mM stock solution. The chemical structure of MS13 and curcumin are shown in Figure 1.

### Cytotoxicity and Anti-Proliferative Assay

The cytotoxicity and anti-proliferative assay were performed using the MTT assay described by Mosmann (Mosmann, 2000) with slight modifications. U-87 MG, SH-SY5Y, and WRL-68 cells were seeded in 96 flat-bottomed tissue culture plate (Nunc^TM^, Roskilde, Denmark) at seeding concentrations of 8 × 10^4^ cells/ml and 10 × 10^4^ cells/ml, respectively. The plates were incubated in a humidified incubator with 5% CO_2_ at 37°C for 24 h to allow the cells to attach to the bottom of the wells. Subsequently, the media were aspirated out and replaced with 100 μl of MS13 or curcumin at their respective concentrations, i.e., 1.5625, 3.125, 6.25, 12.5, 25, 50, and 100 μM. Curcumin serves as positive control, while 0.2% DMSO serves as a negative control. The cells were treated for 72 h for dose-dependent cytotoxicity assay and 24, 48, and 72 h for dose- and time-dependent anti-proliferative assay. Following each treatment, the media containing the compounds were aspirated followed by the addition of 100 μl of 0.5 mg/ml of [3-(4,5dimethylthiazol-2-yl)-2,5-diphenyltetrazolium bromide] (MTT). After 4 h, the MTT solution in each well was removed, and 100 μl of DMSO was added to solubilize the purple-colored formazan product. The absorbance was read at 570 and 640 nm using a microplate reader (BioTek EON^TM^ Microplate Spectrophotometers, Fisher Scientific, United States). The absorbance reading was corrected to background absorbance, and average reading was taken from three independent experiments. The cell viability in percentage was calculated as follows:

(1) $$\mathrm{Cell}\mathrm{viability}\left(\%\right)=\frac{\mathrm{Average}\,\mathrm{absorbance}\,\mathrm{of}\,\mathrm{treated}\,\mathrm{cells}}{\mathrm{Average}\,\mathrm{absorbance}\,\mathrm{of}\,\mathrm{untreated}\,\mathrm{cells}\,\left(\mathrm{control}\right)}\times 100\%$$

The results were analyzed using GraphPad Prism version 8.0.2 for Windows (GraphPad Software, La Jolla, CA, United States). The half-maximal concentration (EC_50_) for each biological replicate was determined using log-response curve constructed. Overall EC_50_ was calculated from the average EC_50_ of three independent biological replicates.

### Selective Index

The selective index (SI) is the degree of selectivity of the compound tested against cancerous cells, in which values larger than “100” indicates the compound is selective toward cancerous cells and confers minimal toxicity in normal non-malignant cells. It was calculated as follows:

(2) $$\mathrm{Selective}\,\mathrm{index}=\frac{{\text{EC}}_{50}\,\mathrm{of}\,\mathrm{WRL}-68}{{\text{EC}}_{50}\,\mathrm{of}\,U-87\,\mathrm{MG}/\mathrm{SH}-\mathrm{SY5Y}}\times 100\%$$

### Caspase-3 Colorimetric Assay

The caspase-3 activity in U-87 MG and SH-SY5Y cells upon exposure to MS13 was examined using RayBiotech^®^ Caspase-3 Colorimetric Assay Kit #68Cl-Casp3-S100 (RayBiotech^®^, Inc., United States). The experiment was performed according to the instructions as described by the manufacturer. First, the cells were seeded in 75-cm^2^ tissue culture flasks (SPL Life Sciences, South Korea) and treated with MS13 at two concentrations, i.e., 1 ×EC_50_ and 2 EC_50_ for three time points, i.e., 24, 48, and 72 h. Uninduced control cells were treated with 0.2% DMSO. Following each treatment, the cells were collected and lysed with 50 μl of chilled lysis buffer. The cell suspension was centrifuged, and cell lysate was collected. The protein concentration in the cell lysate was then quantified using the Pierce^TM^ BCA Protein Assay Kit (Thermo Scientific, Pierce Biotechnology, Rockford, IL, United States). A similar protein amount, i.e., 200 μg was used for each treatment condition during the quantification of caspase-3 activity. A total of three independent experiments were performed, and each sample was quantified twice. The fold-change caspase-3 activity was calculated as follows:

(3) Fold-change⁢of⁢caspase-3⁢activity =Absorbance⁢of⁢MS13⁢induced⁢samples⁢at⁢ 405⁢nmAbsorbance⁢of⁢uninduced⁢control⁢at⁢ 405⁢nm

### Bcl-2 Concentration Colorimetric Assay

The bcl-2 concentration in U-87 MG and SH-SY5Y cells upon exposure to MS13 was examined using Invitrogen bcl-2 ELISA kit #BMS244-3 (Thermo Fisher Scientific, Rockford, IL, United States). The experiment was performed as described by the manufacturer. The cells were first seeded and treated in a similar manner described in the *Caspase-3 Colorimetric Assay* section. Upon exposure to each treatment condition, the cells were collected and lysed using the 1 ×lysis buffer provided. The cell suspension was incubated for 60 min at room temperature with gentle shaking. Subsequently, the cell lysate was collected, and the protein concentration was quantified using the Pierce^TM^ BCA Protein Assay Kit. For each condition, 20 μl of the sample with a protein amount of 200 μg was used for the quantification of bcl-2 concentration. A total of three independent experiments were performed, and the bcl-2 concentration of each sample was quantified twice.

### Statistical Analysis

All experiments were conducted in triplicates. The EC_50_ was reported as the mean ± standard error of the mean (SEM) from three biological replicates. Comparison between the datasets was performed using two-way analysis of variance (ANOVA). All statistical analyses were performed using GraphPad Prism version 8.1.2 for Windows (GraphPad Software, La Jolla, CA, United States).

### Proteomic Analysis

#### MS13 Treatment and Cell Lysis

U-87 MG and SH-SY5Y cells were seeded in 75-cm^2^ tissue culture flasks and exposed to 14 and 10 μM of MS13 (treatment group), respectively, to examine the differential protein expression profile. The cells in the control group were treated with 0.2% DMSO. The MS13 treatment period for U-87 MG and SH-SY5Y cells were 24 and 48 h, respectively. A total of three independent experiments were performed. Following treatment, the media were decanted, and the cells were collected, washed twice with cold 1× phosphate buffer saline (PBS, Corning^®^, Mediatech, Manassas, VA, United States) and lysed with 100 μl of RIPA buffer (Pierce Biotechnology, Thermo Fisher Scientific, Rockford, IL, United States) containing 1× phosphatase and protease inhibitor cocktail (Pierce Biotechnology, Thermo Fisher Scientific, Rockford, IL, United States). The cell suspension was homogenized using ultrasonic cell crusher (Scientz, China), and the cell pellet was spun down using a refrigerated centrifuge (Centrifuge 5424 R, Eppendorf, Germany) at 20,000 rpm, 4°C for 15 min. Subsequently, the cell lysate was collected and measured for its protein concentration using the Pierce^TM^ BCA Protein Assay Kit.

#### In-Solution Digestion

At 1 mg/ml, 60 μg of protein sample collected from each condition was subjected to in-solution digestion. Briefly, 25 μl of 100 mM ammonium bicarbonate (ABC, EMD Millipore, Germany), 25 μl of tetrafluoroethylene (TFE), and 1 μl of 200 mM dithiothreitol (DTT, EMD Millipore, Germany) were added to each protein sample. The sample mixture was vortexed briefly and heated at 90°C. After 1 h, the sample was left to cool until room temperature, followed by the addition of 4 μl of 200 mM iodoacetamide (IAM) (GE Healthcare, United Kingdom) to the mixture. The sample was incubated in the dark at room temperature for 1 h. One microliter of DTT was then added to the sample mixture, vortexed to mix well, and incubated in the dark at room temperature for another hour to quench excess IAM present in the sample mixture. After that, dilution of the sample mixture was performed by adding 300 μl of MilliQ water and 100 μl of ABC. Trypsin (Pierce Trypsin Protease, MS Grade, Thermo Fisher Scientific, Rockford, IL, United States) was then added in a protein:substrate ratio of 1:20, and the samples were incubated at 37°C for 24 h. Last, 1 μl of formic acid was added to stop the trypsin reaction. The sample was then dried using centrifugal evaporator CVE-3100 (Eyela, Japan) and desalted through a C18 spin column (Pierce Biotechnology, Thermo Fisher Scientific, Rockford, IL, United States) as described in the manufacturer’s protocol.

#### Liquid Chromatography Tandem Mass Spectrometry (LCMS/MS) Analysis

The dried, desalted peptide samples were reconstituted with 10 μl of 0.1% formic acid prior to being analyzed on nano-ESI-QTOF (Agilent, 65000 iFunnel Q-TOF LC/MS) equipped with Agilent Large Capacity Chip (G4240-62010 300Å-C18) that was equilibrated with 0.1% formic acid (solution A). One microliter of peptide sample was injected and eluted from the column at a flow rate of 500 nl/min with an increasing 5–70% of solution B, i.e., acetonitrile in water with 0.1% formic acid for 40 min. The polarity was set as positive, the capillary voltage at 1,900 V, and fragmenter voltage at 360 V. The drying gas flowed through the column at 325°C with a flow rate of 5.0 L/min. The spectra were then acquired in auto MS/MS mode in a mass range of 110–3,000 (m/z) for MS scan and 50–3,000 (m/z) for the MS/MS scan. The spectra were then analyzed using PEAKS X software (Bioinformatics Solutions Inc., Waterloo, ON, Canada).

#### Total Protein Identification and Label-Free Quantification

Protein identification in both control and treated samples were performed via automated *de novo* sequencing using PEAKS X software. The peptide spectra were matched against *in silico* digested *Homo sapiens* database downloaded from UniProt^1^. A mass shift of 57.02 Da (carbamidomethylation), fragment mass tolerance of 0.1 Da, monoisotopic precursor, and false-discovery rate (FDR) less than 1.0% were set as the search criterion and trypsin as the digestion enzyme. LFQ was then performed on PEAKS Q module embedded in PEAKS X software. The mass error tolerance was set at 20 ppm, and retention time shift tolerance was set at 6.0 min. At FDR less than 1.0%, proteins that passed through significance (−10l gP) = 13 or equivalent to a *p*-value of less than 0.05, with at least one unique peptide, and a ratio less than 0.76 or more than 1.3 were selected as query terms for subsequent bioinformatic analysis. The ratio is defined as the protein intensity of treated sample vs. control sample.

#### Bioinformatic Analysis

All DEPs obtained from the LFQ analysis were first classified into their respective PANTHER protein classes^2^. Subsequently, statistical enrichment analysis for the biological process, molecular function, and cellular component of the DEP against the gene ontology (GO) consortium database^3^ was performed by submitting the DEPs list as query terms into BiNGO plugin (3.0.3) in Cytoscape (3.7.2) environment. The enriched GO terms were filtered from a *p*-value < 0.05 as the statistically significant cutoff upon the right-sided hypergeometric multiple correction testing with Benjamini–Hochberg option. The protein–protein interaction (PPI) network of all DEPs was then constructed from STRING (*S*earch *T*ool for the *R*etrieval of *IN*teracting *G*enes/Proteins)^4^, which maps the DEPs against the *Homo sapiens* database at high confidence score (0.70). To further identify highly connected protein clusters within the network, k-means clustering analysis was performed. Last, the DEPs identified were mapped to the reactome pathways^5^, in which overrepresentation test at FDR < 0.05 as the criterion was performed to select the associated enriched reactome pathways.

## Results

### Determination of Cytotoxicity of MS13 in U-87 MG and SH-SY5Y Cells Using MTT Assay

**FIGURE 2:**
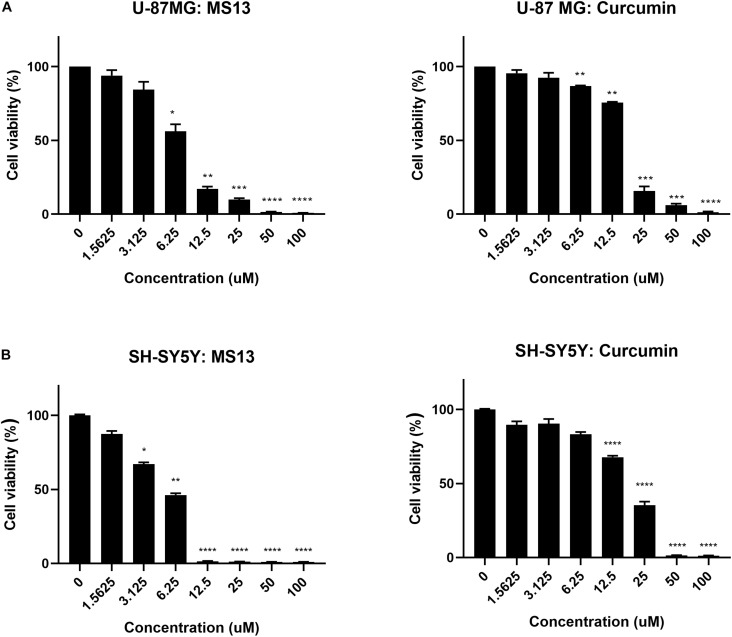
The cell viability in percentage (%) of **(A)** U-87 MG glioblastoma and **(B)** SH-SY5Y neuroblastoma cells upon treatment with MS13 and curcumin. The concentration (ranged from 0 to 100 μM) of MS13 or curcumin was log10 transformed. The cell viability of both cells decreased as the concentration of MS13 or curcumin increased. Results are expressed as the average of percentage of cell viability. Error bars represent mean ± standard error mean. The experiments were performed in triplicates and results were compared between three biological replicates. Statistical analysis was performed using analysis of variance (ANOVA), comparing cell viability of each concentration with the untreated sample. Asterisks indicate the difference in the statistical significance of the mean values between treated and untreated cells. *p < 0.05; **p < 0.01; ***p < 0.001; ****p < 0.0001.

The cytotoxicity of MS13 against human glioblastoma U-87 MG and SH-SY5Y cells were assessed by examining the cell viability at 72 h. Our results showed that MS13 induced a potent dose-dependent cytotoxic effect on U-87 MG and SH-SY5Y cells (Figure 2). Table 1 depicted a comparison of the EC_50_ value of MS13 and curcumin on both SH-SY5Y cells. With an EC_50_ value of approximately 1.4 times lower (U-87 MG: 6.78 ± 1.04 μM; SH-SY5Y: 4.72 ± 1.05 μM), MS13 was more potent toward SH-SY5Y cells than that of U-87 MG cells (*p* < 0.05). The EC_50_ value of curcumin against both cell lines was about 2.5–3.5 times higher than that of MS13 (U-87 MG: 16.39 ± 1.04 μM; SH-SY5Y: 16.52 ± 1.05 μM). Additionally, both MS13 and curcumin recorded an SI value of higher than 100, indicating that these compounds are selective toward human glioblastoma and neuroblastoma cells with minimal toxicity toward human normal cells (Table 1).

**TABLE 1:** Half maximal effective concentration (EC_50_) of MS13 and curcumin against U-87 MG, SH-SY5Y and WRL-68 cells, and their respective selective index.

Compound	EC_50_ (mean ± SEM) (μM)^1^			Selective index (SI)	
		
	U-87 MG	SH-SY5Y	WRL-68	U-87 MG	SH-SY5Y
MS13	6.78 ± 1.04	4.72 ± 1.05	12.81 ± 1.10	189	271
Curcumin	16.39 ± 1.04	16.52 ± 1.07	29.51 ± 1.11	180	179

*^1^EC_50_ values are presented as mean ± standard error mean from three biological replicates.*

### Determination of Anti-Proliferative Effect of MS13 in U-87 MG and SH-SY5Y Cells Using MTT Assay

**FIGURE 3:**
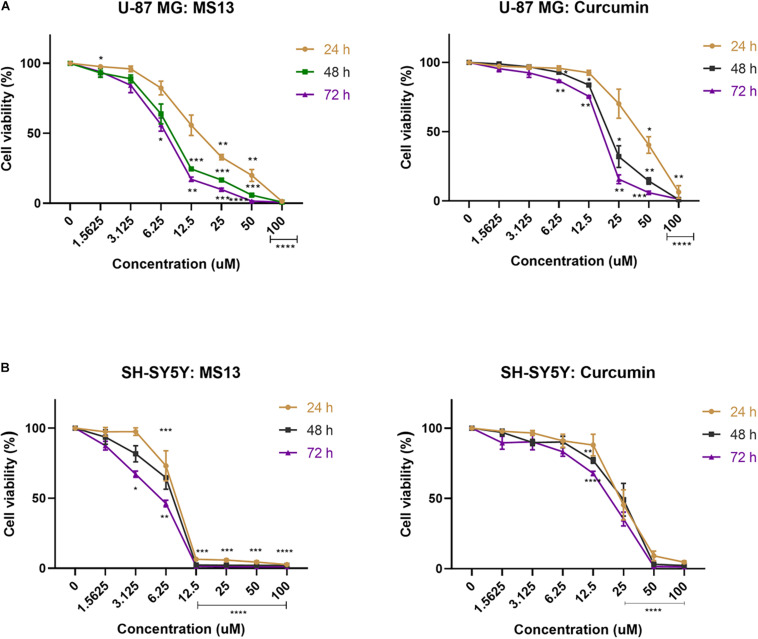
The anti-proliferative effect of MS13 and curcumin on **(A)** U-87 MG glioblastoma and **(B)** SH-SY5Y neuroblastoma cells at 24, 48, and 72 h. Results are expressed as the average of cell viability in percentage (%) against concentration (μM). Error bars represent mean ± standard error mean. All experiments were performed in triplicates and results were compared between three biological replicates. Statistical analysis was performed using ANOVA, comparing cell viability of each concentration with the untreated sample. Asterisks indicate the difference in the statistical significance of the mean values between treated and untreated cells. *p < 0.05; **p < 0.01; ***p < 0.001; ****p < 0.0001.

The anti-proliferative effects of MS13 and curcumin were investigated by measuring the cell viability of U-87 MG cells upon exposure for 24, 48, and 72 h. As shown in Figure 3, MS13 demonstrated dose- and time-dependent anti-proliferative effects on U-87 MG and SH-SY5Y cells. By referring to Table 2, the EC_50_ value of MS13 in U-87 MG cells decreased significantly by about 50% (*p* < 0.05) as the treatment time increased from 24 to 48 h. More significant growth inhibition with a decrease in EC_50_ value to 6.78 μM (*p* < 0.01) was observed when the incubation time increased further to 72 h. Similarly, a progressive reduction in EC_50_ value was observed in SH-SY5Y cells upon MS13 treatment following prolonged treatment time to 72 h. A significant reduction (*p* < 0.01) in EC_50_ value was also observed in these cells following MS13 treatment for 72 h. On the contrary, the cell viability for both U-87 MG and SH-SY5Y remained high following curcumin treatment. The EC_50_ of curcumin were approximately two to four times higher than that of MS13 at all three time-points.

**TABLE 2:** Half maximal effective concentration (EC_50_) of MS13 and curcumin against U-87 MG and SH-SY5Y cells at 24, 48, and 72 h.

Compound/cell line	EC_50_ (mean ± SEM) (μM)^1^		
	
	24 h	48 h	72 h
**MS13**			
U-87 MG	15.93 ± 1.06	8.13 ± 1.05*	6.78 ± 1.04**
SH-SY5Y	7.36 ± 1.05	6.84 ± 1.06	4.72 ± 1.05*
**Curcumin**			
U-87 MG	38.80 ± 1.06	20.45 ± 1.06*	16.39 ± 1.04
SH-SY5Y	22.54 ± 1.06	21.39 ± 1.08	16.52 ± 1.07

^1^EC_50_ values are presented as mean ± standard error mean from three biological replicates. *p < 0.05; **p < 0.01.

### Determination of Apoptosis Activity Using Caspase-3 and bcl-2 Colorimetric Assay

**FIGURE 4:**
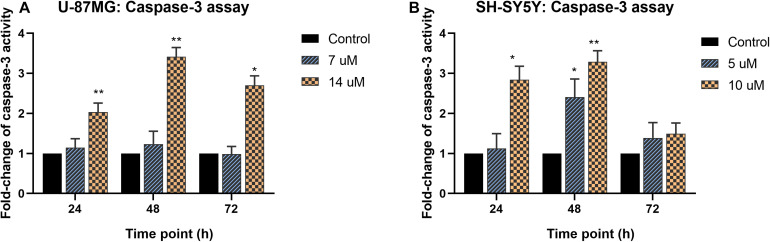
Fold-change of caspase-3 activity in MS13-treated **(A)** U-87 MG and **(B)** SH-SY5Y cells. The caspase-3 activity was measured relative to control (0.2% DMSO) treatment at 24, 48, and 72 h. All experiments were performed in triplicates and results were compared between three biological replicates. Statistical analysis was performed using ANOVA, comparing the caspase-3 activity upon treatment with each MS13 dose at each time point to the respective untreated control sample. Indication of asterisks: *p < 0.05; **p < 0.01.

The caspase-3 activity and bcl-2 concentration in MS13-treated U-87 MG cells were quantified using colorimetric assays, and the cell morphology upon MS13 treatment is shown in Supplementary Figures 1, 2. Our result revealed a significant increase in caspase-3 activity when U-87 MG cells were exposed to 14 μM MS13 for 24, 48, and 72 h but peaked at 48 h with a fold-change of 3.4 in a dose-dependent manner. However, no significant increase was noted following the treatment with 7 μM MS13 for the same period (Figure 4A). On the other hand, the caspase-3 activity in SH-SY5Y cells increased significantly at fold-change 2.8 and peaked at 3.3 following treatment with 10 μM of MS13 for 24 and 48 h, respectively (Figure 4B). No significant increase in the activity was noted in cells treated with 5 μM of MS13 for 24 h, although the activity was found to increase significantly to a fold change of 2.4 as the treatment time prolonged to 48 h. Nevertheless, a decrease in caspase-3 activity was noted with a fold change of less than 1.5 after treatment with 5 or 10 μM for 72 h.

**FIGURE 5:**
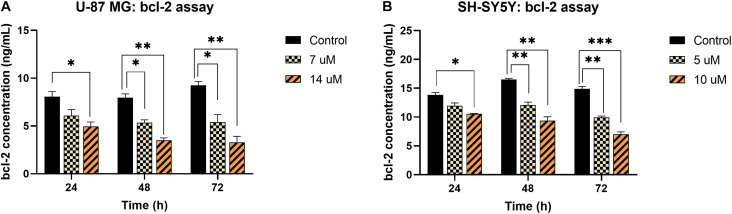
bcl-2 concentration in MS13-treated **(A)** U-87 MG and **(B)** SH-SY5Y cells following 24, 48, and 72 h treatment. All experiments were performed in triplicates and results were compared between three biological replicates. Statistical analysis was performed using ANOVA, comparing the bcl-2 concentration upon treatment with each MS13 dose at each time point to the respective untreated control sample. Indication of asterisks: *p < 0.05; **p < 0.01; ***p < 0.001.

Contrarily, the bcl-2 concentration in both U-87 MG and SH-SY5Y cells was found to decrease significantly upon exposure to MS13. In U-87 MG cells, reduction in bcl-2 was noted following treatment with 7 μM, and the reduction was more prominent when treated with 14 μM of MS13 for 24, 48, and 72 h (Figure 5A). Similarly, the bcl-2 concentration in SH-SY5Y cells reduced significantly following treatment with 5 μM of MS13, and a greater reduction of bcl-2 concentration was observed when the concentration of MS13 was increased to 10 μM and exposed for 24, 48, and 72 h (Figure 5B). These data suggest that MS13 induces apoptosis via caspase-3 and mitochondrial-dependent pathway in U-87 MG cells in a dose- and time-dependent manner.

### Analysis of DEPs in MS13-Treated U-87 MG and SH-SY5Y Cells via Shotgun Proteomic Approach

The differential protein expression profile in U-87 MG and SH-SY5Y cells following MS13 treatment was investigated via label-free shotgun proteomic approach. The optimum dosage and time selected for U-87 MG and SH-SY5Y cells were 14 μM (2 × EC_50_) of MS13 for 24 h and 10 μM (2 × EC_50_) of MS13 for 48 h, respectively. These parameters were selected based on the data analysis obtained from cytotoxicity, anti-proliferative, and apoptosis assays, whereby the cells were undergoing apoptosis with at least 50% cell viability upon exposure to these treatment conditions. A total of 406 and 349 proteins were identified in U-87 MG cells in the control and treatment group, respectively. Among these proteins, a total of 30 DEPs comprising of nine upregulated and 21 downregulated proteins were identified in MS13-treated U-87 MG cells. In SH-SY5Y cells, 437 and 435 proteins were determined in the control and treatment group, respectively, and 38 DEPs that comprise of seven upregulated and 31 downregulated proteins were identified.

#### Gene Ontology: Functional Annotation

**FIGURE 6:**
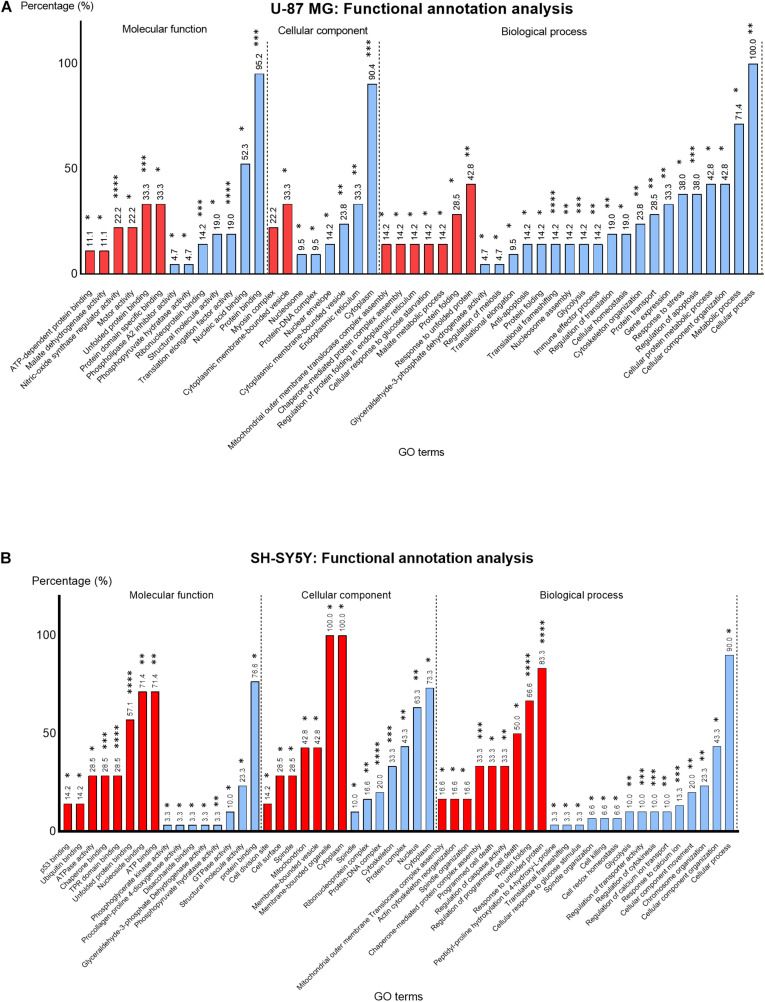
Percentage (%) of up- (red) and downregulated (blue) proteins identified in MS13-treated **(A)** U-87 MG and **(B)** SH-SY5Y cells annotated to each statistically enriched GO term for functional categories “Molecular function,” “Cellular component,” and “Biological process.” The number above each bar represents the percentage of protein of the respective GO terms while the asterisks indicate the false-discovery rate (FDR) (*p*-value) of each GO term upon multiple correction statistical test using the Benjamini–Hochberg procedure. *p < 0.05; **p < 0.01; ***p < 0.001; ****p < 0.0001.

The DEPs lists identified in U-87 MG and SH-SY5Y cells were uploaded to BiNGO plugin in Cytoscape for functional annotation analysis. The enriched GO terms in each functional category, namely, the molecular function, cellular component, and biological process were identified (Figure 6).

In U-87 MG cells, the GO terms “Nitric-oxide synthase regulator activity” (22.2%) (*p* < 0.0001) and “Unfolded protein binding” (33.3%) (*p* < 0.001) appeared to be the most significantly enriched GO terms among the upregulated proteins, while the “Translation elongation factor activity” (19%) (*p* < 0.0001), “Protein binding” (95.2%) (*p* < 0.001), and “Ribonucleoprotein binding” (14.2%) (*p* < 0.001) were the most significantly enriched GO terms among the downregulated proteins in the molecular function category (Figure 6B). On the other hand, “Unfolded protein binding” (67.1%) (*p* < 0.0001), “ATPase activity” (28.5%) (*p* < 0.0001), and “TPR domain binding” (28.5%) (*p* < 0.001) were the most significantly enriched molecular function GO terms annotated by upregulated proteins in SH-SY5Y cells (Figure 6B). Although the majority of the downregulated proteins were associated with protein binding (76.6%), the association was not highly significant (*p* < 0.05).

Based on the cellular component category, the most significantly enriched GO term annotated by the upregulated proteins in U-87 MG cells was “Myosin complex” (22.2%) (*p* < 0.01). However, the majority of the downregulated proteins (90.4%) were associated with “Cytoplasm” and noted as the top most significantly enriched (*p* < 0.001) GO term, while all (100%) upregulated proteins in the SH-SY5Y cells were associated with “Cytoplasm” and “Membrane-bounded organelle,” whereas the majority of the downregulated proteins (73.3%) were associated with “Cytoplasm.” Nonetheless, “Protein–DNA complex” (16.6%) (*p* < 0.0001) and “Cytoskeleton” (20.0%) (*p* < 0.001) were the two most significantly enriched cellular component GO terms annotated by downregulated proteins.

The most enriched biological process GO term among the upregulated proteins in U-87 MG cells was “Response to unfolded protein” (42.8%) (*p* < 0.01). However, the “Translational frameshifting” (14.2%) (*p* < 0.0001), “Regulation of apoptosis” (38.0%) (*p* < 0.001), and “Glycolysis” (14.2%) (*p* < 0.001) were the most significant GO terms identified among the downregulated proteins. Although all the downregulated proteins in this category were associated with “cellular process” (100%) and a large proportion with “metabolic process” (71.4%), the statistical analysis indicated the association was not highly significant. Similarly, a majority of the upregulated proteins in SH-SY5Y cells were associated with “Response to unfolded protein” (83.3%) and “Protein folding” (66.6%), whereby these GO terms were highly significant (*p* < 0.0001). Additionally, “Chaperone-mediated protein complex assembly” (33.3%) (*p* < 0.001) was another highly significant biological process GO term annotated by these proteins. On the contrary, “Response to calcium ion” (13.3%) (*p* < 0.001), “Regulation of cytokinesis” (10.0%) (*p* < 0.001), and “Regulation of transporter activity” (10.0%) (*p* < 0.001) were highly significant enriched biological process GO terms annotated by downregulated proteins. Although majority of these proteins were associated with the cellular process (90.0%), the statistical analysis revealed that the association was not highly significant.

#### Protein Identification and Classification

The DEPs identified in MS13-treated U-87 MG and SH-SY5Y cells with a ratio of more than 1.3 and less than 0.76 were grouped as upregulated and downregulated proteins, respectively. These DEPs were then classified into their respective protein classes by mapping them into the PANTHER classification system. Nine of the DEPs, including heat shock protein HSP 90-alpha (HSP90AA1), HSP90AB1, tubulin beta chain (TUBB), glyceraldehyde 3-phosphate dehydrogenase (GAPDH), alpha-enolase (ENO1), histone H2AX (H2AFX), heterogenous nuclear ribonucleoprotein K (HNRNPK), eukaryotic translation initiation factor 5A-1 (EIF5A), and SET were commonly found in MS13-treated U-87 MG and SH-SY5Y cells.

By referring to Supplementary Table 1, the upregulated proteins identified in MS13-treated U-87 MG cells were grouped into four protein classes. The top protein classes were chaperone and cytoskeletal-binding protein, each comprised 44% (4/9) and 33% (3/9) of the upregulated proteins, while histone and metabolic enzyme comprised 11.1% (1/9) of the proteins. The downregulated proteins were classified into nine protein classes. The top protein class identified was the RNA-binding protein that comprised 23.8% (5/21) of the downregulated proteins followed by metabolic enzyme and chaperone, wherein each comprised 19.0% (4/21) of the proteins. The remaining of the protein classes grouped by downregulated proteins included histone and cytoskeletal protein, wherein each made up of 9.5% (2/21) of the proteins, and ribosomal protein, calcium-binding protein, enzyme modulator, and cytoskeletal binding protein, wherein each constituted 4.8% (1/21) of the proteins.

On the other hand, the upregulated proteins in MS13-treated SH-SY5Y cells were grouped into three protein classes (Supplementary Table 2). Chaperone was the top protein class, which comprised 71.4% (5/7) of the proteins. The remaining protein classes were metabolic enzymes and cytoskeletal-binding proteins, and each consisted 14.3% (1/7) of the upregulated proteins. A total of 31 downregulated proteins were determined and classified into 12 protein classes. The top protein classes were histones and cytoskeletal proteins that comprised 19.4% (6/31) of the downregulated proteins. This was followed by metabolic enzymes, RNA-binding protein, calcium-binding protein, and enzyme modulator, which comprised 16.1% (5/31), 12.9% (4/31), 9.7% (3/31), and 6.5% (2/31) of the proteins. However, 3.2% (1/31) of the downregulated proteins were ribosomal protein, cytoskeletal-binding protein, cell adhesion molecule, cation transporter, and chaperone.

#### Protein–Protein Interaction Network

**FIGURE 7:**
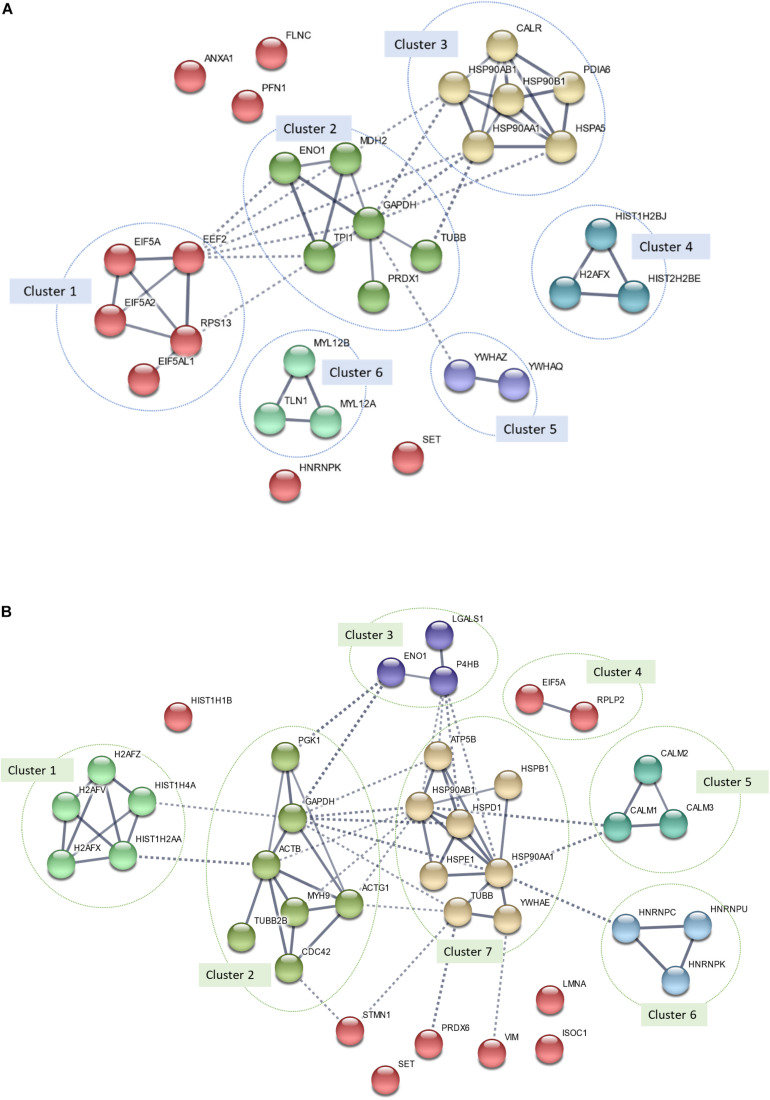
Protein–protein interaction (PPI) network of all differentially expressed proteins (DEPs) identified in MS13-treated **(A)** U-87 MG (*p*-value = 2.64 × 10^–14^) and **(B)** SH-SY5Y (*p*-value = 1.84 × 10^–14^) cells. The nodes represent proteins, wherein the nodes in the same cluster demonstrated the same color. The lines connecting the nodes indicate the association between the proteins. The thicker the line, the higher the degree of confidence prediction of the interaction. Dashed lines represent inter-cluster between the highly connected clusters.

The PPI network of all DEPs identified in MS13-treated U-87 MG (*p* = 2.64 × 10^14^) and SH-SY5Y (*p* = 1.84 × 10^–14^) cells are presented in Figures 7A,B, respectively. A total of 30 nodes and 46 edges were identified in the PPI network of U-87 MG cells, while 38 nodes and 68 edges were found in SH-SY5Y cells. A significant interaction between DEPs with average local clustering of 0.663 and 0.604 for U-87 MG and SH-SY5Y cells, respectively, were determined. Both networks also revealed that the DEPs with similar functional roles were grouped into a cluster, and inter-cluster interactions were clearly displayed.

In MS13-treated U-87 MG cells, a total of six highly connected clusters were generated from k-means clustering analysis. Among the six clusters, clusters 1, 2, and 3 were highly connected. Cluster 1 was made up of RNA-binding proteins (e.g., EIF5A, EIF5A2, EIF5AL1, and EEF2) and ribosomal proteins (e.g., RPS13). Metabolic enzymes (e.g., ENO1, MDH2, GAPDH, TPI1, and PRDX1) and cytoskeletal protein (e.g., TUBB) made up Cluster 2. Chaperones including CALR, PDIA6, and heat shock proteins (e.g., HSP90AB1, HSP90AA1, HSP90B1, and HSPA5) made up cluster 3. Clusters 4, 5, and 6 each were made up of histones (e.g., HIST1H2BB, H2AFX, and HIST1H2BJ), 14-3-3 proteins (e.g., YWHAZ and YWHAQ), and cytoskeletal proteins (e.g., MYL12B, MYL12A, and TLN1). The remaining un-clustered proteins were ANXA1, PFN1, FLNC, SET, and HNRNPK.

In MS13-treated SH-SY5Y cells, a total of seven clusters were made up of DEPs with similar functional roles identified. The first cluster was made up of histones (e.g., H2AFZ, H2AFV, HIST1H4A, HIST1H2AA, and H2AFX). Cluster 2 consists of cytoskeletal proteins (e.g., ACTB, ACTG1, MYH9, TUBB2B, and CDC42) and metabolic enzymes (e.g., PGK1 and GAPDH), while Cluster 3 was made up of metabolic enzymes (e.g., ENO1 and P4HB) and cell adhesion molecules (e.g., LGALS1). Cluster 4 was made up of RNA-binding proteins (EIF5A) and ribosomal proteins (RPLP2). Clusters 5 and 6 each consisted of calmodulins (CALM1, CALM2, and CALM3) and RNA-binding proteins (HNRNPK, HNRNPU, and HNRNPK). Chaperones (e.g., HSPB1, HSP90AB1, HSP90AA1, HSPD1, HSPE1, and YWHAE), cation transporters (e.g., ATP5B), and cytoskeletal proteins (e.g., TUBB) made up Cluster 7. The remaining unclustered proteins were HIST1H1B, STMN1, SET, PRDX6, VIM, LMNA, and ISOC1.

#### Pathway Analysis

All DEPs identified in MS13-treated U-87 MG and SH-SY5Y cells were then mapped into the reactome pathway database to identify the possible pathways modulated by MS13. An overrepresentation analysis was performed. “Glycolysis” and “Post-translational protein modification” were the two common pathways identified in both U-87 MG and SH-SY5Y cells. The rest of the top enriched pathways identified in U-87 MG cells were “G2/M DNA damage checkpoints,” “Axon guidance,” and “Intrinsic pathway for apoptosis,” while for SH-SY5Y cells, “Rho GTPase-activated IQGAPs,” “Cellular responses to stress,” and “Apoptotic execution phase” pathways were identified. The DEPs mapped to each pathway are tabulated in Table 3.

**TABLE 3:** Enriched reactome pathways mapped by all DEPs identified in MS13-treated U-87 MG and SH-SY5Y cells.

Pathway ID	Pathway name	Protein^1^	FDR^2^
**U-87 MG**			
R-HSA-69481	G2/M DNA damage checkpoints	H2AFX*; YWHAQ; HIST2H2BE; HIST1H2BJ; YWHAZ	1.01 × 10^–3^
R-HSA-422475	Axon guidance	HSP90AA1*; HSP90AB1*; MYL12B; MYL12A; PFN1; TLN1	2.36 × 10^–3^
R-HSA-70171	Glycolysis	ENO1*; GAPDH*; TPI1	3.59 × 10^–3^
R-HSA-109606	Intrinsic Pathway for Apoptosis	YWHAQ; YWHAZ	1.79 × 10^–2^
R-HSA-597592	Post-translational protein modification	HIST2H2BE; HIST1H2BJ; EIF5A2; EEF2; PDIA6; EIF5A*; HSP90B1; HNRNPK*; CALR	1.97 × 10^–2^
**SH-SY5Y**			
R-HSA-194315	Rho GTPase activates IQGAPs	TUBB2B; TUBB*; ACTB; ACTG1; CALM1; CDC42	1.24 × 10^–5^
R-HSA-2262752	Cellular responses to stress	HSP90AA1*; HSP90AB1*; HIST1H4A; HIST1H1B; H2AFX*; H2AFZ; H2AFV; PRDX6; P4HB; YWHAE	1.87 × 10^–5^
R-HSA-75153	Apoptotic execution phase	VIM; LMNA; HIST1H1B	3.50 × 10^–3^
R-HSA-70171	Glycolysis	ENO1*; GAPDH*; PGK1	8.29 × 10^–3^
R-HSA-392499	Post-translational protein modification	EIF5A*; HNRNPC; HNRNPK*; HIST1H4A; HIST1H2AA; TUBB2B; P4HB; CALM1	4.54 × 10^–2^

*^1^Proteins in blue and purple represents up- and downregulated proteins, respectively.*

^2^FDR, false discovery rate, corrected using Benjamini–Hochberg multiple testing procedure.

*Common differentially expressed proteins (DEPs) identified in both U-87 MG and SH-SY5Y cells.

## Discussion

The present study showed that the viability of U-87 MG and SH-SY5Y cells decreased as the concentration of curcumin and MS13 increased. Notably, MS13 induced more potent cytotoxicity and anti-proliferative effects, with an EC_50_ value approximately 2.4- and 3.5-fold lower than that of curcumin in U-87 MG and SH-SY5Y cells, respectively. Additionally, the reduction in cell viability follows a dose- and time-dependent manner. The selectivity of MS13 against cancerous cells was verified by comparing the EC_50_ of MS13 on cancerous cells against normal hepatocytes. A higher SI value depicted by MS13 further implies that MS13 is not only being a more toxic compound, but it is also selective toward cancerous cells compared with curcumin. However, to further compare the selectivity of MS13 between brain cancerous and normal cells, the cytotoxicity effect of MS13 on normal glial and neuronal cells warrants further investigation in the future. The chemical modification in the central spacer region of curcumin may account for the enhanced cytotoxicity and anti-proliferative effects as displayed by MS13. Liang et al. (2009) revealed that the weak pharmacokinetic profiles of curcumin were due to the high susceptibility of OH– in the central spacer β-diketone moiety toward nucleophilic attack. The curcumin analog that lacks the β-diketone moiety is more stable and often exhibits enhanced biological activities than those of curcumin (Liang et al., 2009). Additionally, MS13 also demonstrated a higher degree of cytotoxicity with a lower EC_50_ value compared with curcumin on several cancer cells, including nasopharyngeal (Liang et al., 2009), colorectal (Cen et al., 2009; Liang et al., 2009), prostate (Citalingam et al., 2015), cervical (Paulraj et al., 2015), and gastric (Yoshida et al., 2018) cancer cells. The present findings are consistent with previous studies indicating that the removal of the β-diketone moiety may yield compounds that exhibit improved cytotoxicity and anti-proliferative effects over its parent compound.

The cytotoxicity and anti-proliferative effects induced by MS13 were more potent in neuroblastoma SH-SY5Y cells compared with human glioblastoma U-87 MG cells. Glioblastoma originates from astrocytes, while neuroblastoma arises from neuroblasts. Kim et al. (2015) have reported that U-87 MG cells adopted a specific carbohydrate metabolism and allowed these cells to possess a higher metabolic capacity; thus, they divide more rapidly, compared with SH-SY5Y cells. Additionally, the molecular disparity between U-87 MG and SH-SY5Y cells may also account for the difference in sensitivity toward MS13 treatment, wherein the former cells documented chromosomal deletions (Miyakawa et al., 2000), while the latter has been reported to harbor a complete set of chromosomes (Yusuf et al., 2013). Collectively, it is suggested that both biological and molecular characteristics are responsible for the sensitivity of U-87 MG and SH-SY5Y cells toward MS13 treatment, with a therapeutic advantage against neuroblastoma cells by MS13.

Apoptosis is a preferable means exploited by anti-neoplastic agents during chemotherapy as it removes potentially harmful cells without causing extensive inflammatory-associated damage in the body. In the present study, caspase-3 activity and concentration of bcl-2 were determined in U-87 MG and SH-SY5Y cells following treatment with 1 × EC_50_ (U-87 MG: 7 μM; SH-SY5Y: 5 μM) and 2 × EC_50_ (U-87 MG: 14 μM; SH-SY5Y: 10 μM) of MS13 at three-time points, 24, 48, and 72 h. Caspase-3, which plays a critical role in executing apoptosis, is an effector molecule activated by caspase-8, -9, and -10. Given that caspase-3 regulates the occurrence of the downstream biochemical and morphological hallmarks (Boland et al., 2013), it is introduced as a potential druggable molecular target for the chemotherapeutic agent. Our present findings revealed that caspase-3 activity increased in a dose-dependent manner and peaked at 48 h in both MS13-treated U-87 MG and SH-SY5Y cells. Supportively, FLLL-11, which has an identical chemical structure as MS13 and diarylpentanoid B19, induced apoptosis in human colorectal cancer (Cen et al., 2009) and ovarian cancer (HO8910) (Qu et al., 2013) cells via activation of caspase-3, respectively. Bcl-2- is an anti-apoptotic protein in the BCL-2 family that regulates the mitochondrial-dependent apoptosis pathway via the inhibition of membrane permeabilization activity and suppression of the release of cytochrome c (Kalkavan and Green, 2018). Given the positive correlation between bcl-2 overexpression and tumorigenesis, compounds that downregulate bcl-2 may confer a therapeutic advantage in treating cancers (Adams and Cory, 2018). We demonstrated a progressive reduction in bcl-2 concentration in both U-87 MG and SH-SY5Y cells upon exposure to MS13, and the reduction is in a dose- and time-dependent manner. Earlier studies had also noted similar observations when melanoma cells were treated with diarylpentanoid (DM-1) (Faião-Flores et al., 2013) and hepatocellular carcinoma cells with EF24 (Liu et al., 2012). These findings suggest that MS13 induces mitochondrial-dependent apoptosis in both U-87 MG and SH-SY5Y cells.

To date, no previous studies have been conducted to investigate the anti-cancer effects of MS13 via the shotgun proteomics approach. Therefore, the large-scale proteomics analysis performed in our current study shed a light on the protein expression profile of U-87 MG and SH-SY5Y cells following MS13 treatment. Enrichment analysis of these DEPs using functional annotation demonstrated several most significantly enriched GO terms in the cellular component, biological process, and molecular function categories. The most significantly enriched GO terms commonly identified in both cell lines were the unfolded protein binding categorized under molecular function, and response to unfolded protein and glycolysis under the biological process. Notably, GO terms such as cytoplasm, translational frameshifting, regulation of apoptosis, nitric oxide synthase regulator activity, protein binding, translation elongation factor activity, and ribonucleoprotein binding were found highly significantly enriched in U-87 MG cells; while cytoskeleton and protein–DNA complex, ATPase activity and TPR domain binding, protein folding, chaperone-mediated protein complex assembly, response to calcium ion, regulation of cytokinesis, and regulation of transporter activity were observed in SH-SY5Y cells. These findings suggest that MS13 induces cytotoxicity, anti-proliferation, and apoptosis in U-87 MG and SH-SY5Y cells through DEPs associated with cellular and metabolic processes, cellular responses, transcriptional and translational regulation, induction of cellular stress, and transporter activity.

We further investigate the interaction of all DEPs identified in MS13-treated U-87 MG and SH-SY5Y cells by performing PPI network analysis, which revealed the functional network. Furthermore, integration of DEPs from PPI network analysis with the top five reactome pathways of both cell lines revealed that proteins in the clusters also mapped into the pathways underscores the importance of these pathways in determining the anti-cancer effects of MS13 in U-87 MG and SH-SY5Y cells. The common DEPs that were mapped in the pathways include GAPDH, ENO1, HSP90AA1, heat shock protein HSP 90-beta (HSP90BAB1), EIF5A, HNRNPK, and H2AFX. Additionally, TUBB was the common DEPs that mapped only to the enriched pathway in SH-SY5Y but not U-87 MG cells, while Protein SET (SET) did not map into any of the enriched pathways. We will focus on the biological significance of these proteins in the respective clusters and associated pathways.

Glyceraldehyde 3-phosphate dehydrogenase and ENO1, as mutually downregulated proteins identified in both U-87 MG and SH-SY5Y cells, were mapped to one of the mutual pathways “Glycolysis” for both cell lines. The Warburg hypothesis indicated aerobic glycolysis as one of the core metabolic pathways employed by cancer cells to generate energy (Warburg, 1956). Given this hypothesis, targeting the molecules along the glycolytic pathway confers a therapeutic advantage in treating cancer cells by starving these cells to death. GAPDH catalyzes the conversion of glyceraldehyde-3-phosphate (GAP) to 1,3-bisphosphoglycerate (1,3BP) and ENO1 dehydrates 2 phosphoglycerate (2PG) to form phosphoenolpyruvate (PEP). These interconnected glycolytic proteins catalyze the conversion of glucose to generate ATP, NADH, and pyruvate molecules to support the high load of anabolic reaction in cells. Overexpression of these proteins in the secretome of a glioblastoma cell line was associated with the progression of glioblastoma (Gupta et al., 2013). Li et al. (2019) also showed that the growth and the progression of SH-SY5Y cells were significantly reduced following treatment with glycolysis inhibitory peptide. Altogether, the findings imply that MS13 may demonstrate its anti-cancer effects in U-87 MG and SH-SY5Y cells by downregulating GAPDH and ENO1, which disrupts the glycolytic pathway and subsequently results in the reduction of energy production and inhibition of cell proliferation.

The heat shock proteins, HSP90AA1 and HSP90AB1 were found upregulated in both U-87MG and SH-SY5Y cells and mapped to “Axon guidance” and “Cellular responses to stress” pathways, respectively. The key functional role of these cytoplasmic chaperones is to regulate protein homeostasis by maintaining the proteome in a folded and functional state (Calderwood and Murshid, 2017). Hence, the upregulation of heat shock proteins as inducible chaperones is expected as the proteolytic and oxidative insults accumulate. HSP90AA1 and HSP90AB1 are involved in the axon guidance pathway, via an association with the semaphorins and their signaling mechanism. The axon guidance pathway regulates the neural development and migration, wherein semaphorins are one of the protein families that finetune the morphology and motility of various types of cells including those associated with the nervous system (Chedotal et al., 2005). Additionally, both HSP90AA1 and HSP90AB1 also regulate the LIM kinase (LIMK) protein dimerization and prevent the binding of phosphorylated cofilin to the actin molecules (Li et al., 2006). Hence, the upregulation of HSP90AA1 and HSP90BB1 in the current study indicates the ability of MS13 to induce environmental stress and inhibit the actin polymerization activity, leading to the disruption of the cytoskeletal remodeling process. The findings suggest that these proteins may inhibit cell migration, activate cytoprotective measure upon exposure to an increase oxidative or proteolytic stress as induced by MS13 and apoptosis in U-87 MG and SH-SY5Y cells.

The RNA-binding proteins, EIF5A and HNRNPK, were downregulated in U-87 MG and SH-SY5Y cells that mapped to “Post-translational protein modification,” a pathway that significantly imparts a dynamic complexity in proteins, leading to a diverse functional role of the proteins (Krueger and Srivastava, 2006). The EIF5A is a translational factor that is involved in polypeptide chain elongation, while the isoform of EIF5A-EIF5A2- was identified exclusively in U-87 MG cells and is also downregulated in this pathway. Both proteins are activated upon hypusination, a two-step enzymatic post-translational modification process that transform the lysine residue of the translation factors to hypusine and is involved in the regulation of cell growth and proliferation (Mathews and Hershey, 2015). The overexpression of both isoforms was associated with poor prognosis of glioblastoma (Preukschas et al., 2012). The spermidine and deoxyhypusine synthase (DHPS) inhibitor, GC7, inhibits activation of EIF5A and induces apoptosis of neuroblastoma cells via the regulation of the p21/Rb signaling axis (Bandino et al., 2014). Consistently, the knockdown of EIF5A2 inhibits the migration and invasion of non-small cell lung cancer cells (Chen et al., 2018). On the other hand, the HNRNPK regulates activities associated with RNA metabolism, including pre-mRNA processing and mRNA nucleo-cytoplasmic transportation. HNRNPs promote cell proliferation and metastasis by binding to oncogenes, such as *c-myc* at their promoter region (Kim et al., 2003). The overexpression of HNRNPK was associated with neuroblastoma progression (Li et al., 2018), while downregulation of HNRNPK repressed glioblastoma cell proliferation and induced apoptosis (Wu et al., 2020). Collectively, our current finding suggests that downregulation of EIF5A and HNRNPK by MS13 may inhibit cell proliferation and migration as well as induce apoptosis in glioblastoma and neuroblastoma cells.

Histones are essential in packaging the DNA double helix into nucleosomes. H1 proteins are linkers that stabilize histone octamers, i.e., H2, H3, and H4 (Chikhirzhina et al., 2018). The core histone variant, H2AFX, was identified as one of the common DEPs in both cells, and was up- and downregulated in U-87 MG and SH-SY5Y cells, respectively. This protein was mapped to different pathways, “G2/M DNA damage checkpoints” and “Cellular responses to stress” for U-87 MG and SH-SY5Y cells, respectively. The H2AFX is a DNA double-stranded break response protein that accumulates and activates the CHEK2 protein via auto-phosphorylation at the G2/M DNA damage checkpoint. The activated CHEK2 protein inhibits CDC25C phosphatase, stabilizes the p53 protein, and halts the cell cycle progression. Liu et al. (2007) reported that H2AFX may serve as a tumor suppressor protein, and its upregulation sensitizes the gastrointestinal stromal tumor cells to imatinib chemotherapeutic drug treatment. Nevertheless, downregulation of H2AFX may also indicate genomic instability in cells exposed to external stimuli. Supportively, Braunstein et al. (2016) revealed that breast cancer cells with downregulated H2A and H2B were more sensitive toward anthracycline-induced apoptosis. Collectively, the deregulation of H2AFX may disrupt the DNA replication process, DNA damage repair, and chromosomal stability in both cell lines treated with MS13. These findings suggest that H2AFX may interfere with the transcriptional machinery by sensitizing both glioblastoma and neuroblastoma cells toward MS13 treatment, and thus leading to apoptosis.

Besides, the cytoskeletal protein TUBB was found downregulated in U-87 MG and SH-SY5Y cells. Nonetheless, TUBB was only mapped to “Rho GTPase activates IQGAPs” in SH-SY5Y cells but not in U-87 MG cells. The signaling by Rho GTPases regulates cell shape, cell proliferation, cell adhesion, and cell migration. Given that the cytoskeletal proteins, including microtubules, are essential in regulating cellular processes such as cell division, migration, and intracellular transport, the mutations or alterations in the expression and stability of these proteins were associated with cancer progression (Ong et al., 2020). Therefore, tubulins are promising therapeutic targets in treating cancer. Taxanes and vincristines are two most notable chemotherapeutic drugs that were reported to induce cancer cell death by arresting the cells at mitotic phase by targeting microtubules such as TUBB at the mitotic phase (Kavallaris, 2010). As microtubule assembly underpins the structural integrity of cells and facilitate cell cycle progression (Hall, 2009; Chen and Xie, 2018), downregulation of TUBB by MS13 may inhibit cell migration, disrupt cell integrity, and subsequently lead to cell cycle arrest and apoptosis in glioblastoma and neuroblastoma cells.

Protein SET, formerly known as Oncoprotein 17, was downregulated in MS13-treated U-87 MG and SH-SY5Y. Notably, the overexpression of SET has been associated with poor prognosis, high recurrence rate, and chemoresistance (Cristóbal et al., 2015) in several types of cancer including lung (Liu et al., 2015), glioblastoma (He et al., 2016), breast (Huang et al., 2018), and pancreas (Bhutia et al., 2013). By inhibiting the protein phosphatase 2A (PP2A) activity, SET interferes with the tumor suppressor function of PP2A. This will have an impact on cellular processes such as apoptosis, protein synthesis, signal transduction, and stress response. Additionally, SET also interacts with Rac1 (Switzer et al., 2011) and nm23-H1 (Fan et al., 2003) to regulate cancer metastasis and inhibits p53 to promote cancer stemness (Wang et al., 2016). Therefore, serving as a promising target, SET antagonists such as sphingosine FTY720 (Oaks et al., 2013) was shown to induce anti-proliferative and anti-migratory activities in cancers. As SET was downregulated in both U-87 MG and SH-SY5Y cells, MS13 may also induce its anti-cancer effects in both glioblastoma and neuroblastoma by serving as a SET inhibitor.

## Conclusion

The curcumin analog, MS13, demonstrates potent dose-dependent cytotoxicity, and time- and dose-dependent anti-proliferative activity in both U-87 MG and SH-SY5Y cells, over its parent compound, curcumin. MS13 also induces caspase-3 and mitochondrial-dependent apoptosis in both cells. The proteomic analysis of these cells following treatment with MS13 has revealed several common DEPs, i.e., ENO1, HSP90AA1, HSP90AB1, EFI5A, HNRNPK, TUBB, H2AFX, and SET and common top enriched pathways, i.e., “Glycolytic” and “Post-translational protein modification” pathways. Collectively, our current findings suggest that MS13 may induce its cytotoxicity, anti-proliferation, anti-migration, and apoptosis activities in human glioblastoma and neuroblastoma cells via the regulation of DEPs through PPI and specific pathways. These findings derived from proteomics analysis warrants further investigation to uncover the anti-cancer effects of MS13 on these cells in a holistic view.

## Data Availability Statement

The datasets presented in this study can be found in online repositories. The names of the repository/repositories and accession number(s) can be found below: The mass spectrometry proteomics data have been deposited to the ProteomeXchange Consortium via the PRIDE [1] partner repository with the dataset identifiers PXD024565 (Glioblastoma) and PXD024564 (Neuroblastoma).

## Author Contributions

RN was in charge of the conceptualization, methodology, supervision, visualization and writing, reviewing, and editing of the manuscript. PR and IO did the supervision and writing, reviewing, and editing of the manuscript. FA provided the resources. YL was responsible for the investigation, formal analysis, data curation, and preparation, and writing of the original draft. All authors contributed to the article and approved the submitted version.

## Conflict of Interest

The authors declare that the research was conducted in the absence of any commercial or financial relationships that could be construed as a potential conflict of interest.

## Footnotes

1 https://www.uniprot.org/

2 http://www.pantherdb.org/

3 http://geneontology.org/

4 https://string-db.org/

5 https://reactome.org/
