# Supplementary material for: Proteomic Analysis on Anti-Proliferative and Apoptosis Effects of Curcumin Analog, 1,5-bis(4-Hydroxy-3-Methyoxyphenyl)-1,4-Pentadiene-3-One-Treated Human Glioblastoma and Neuroblastoma Cells
Source: Front Mol Biosci. 2021 Apr 30;8:645856. doi: 10.3389/fmolb.2021.645856 (PMC8119891; doi:10.3389/fmolb.2021.645856)
Supplement: Supplementary file 1 [file Table_1.docx]

Supplementary Material


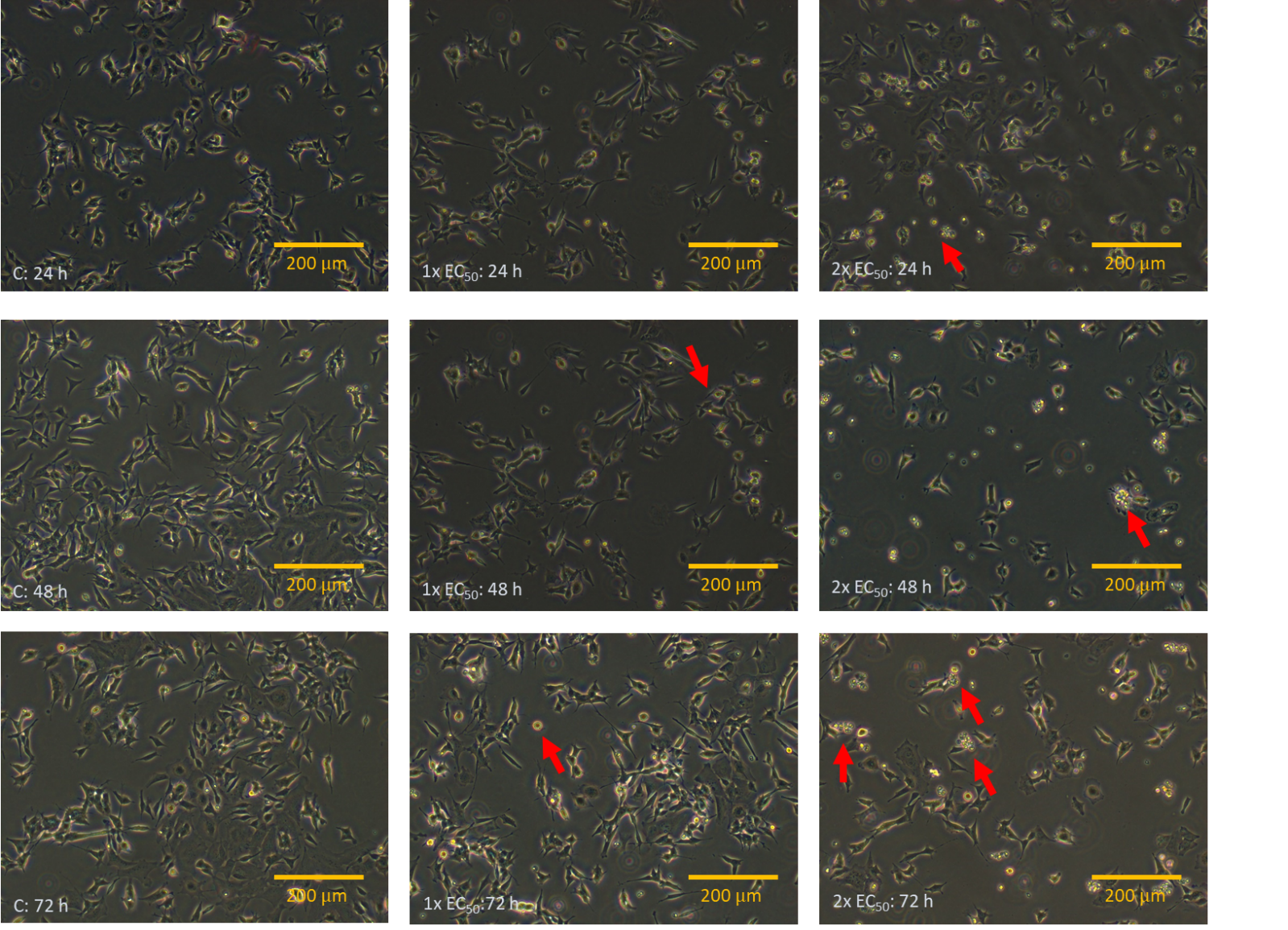


Supplementary Figure 1: SH-SY5Y cell morphology following MS13 treatment at concentration 1 $\times$ EC_50_ (5 $\mu$M) and 2 $\times$ EC_50_ (10 $\mu$M) compared with control (C) at treatment point of 24, 48 and 72 hours. The cell image was taken at 100× magnification. Red arrows indicate floating cells observed in MS13 treatment flask. These phase contrast cell images were captured using inverted microscope CKX14-A22 (Olympus, Japan).


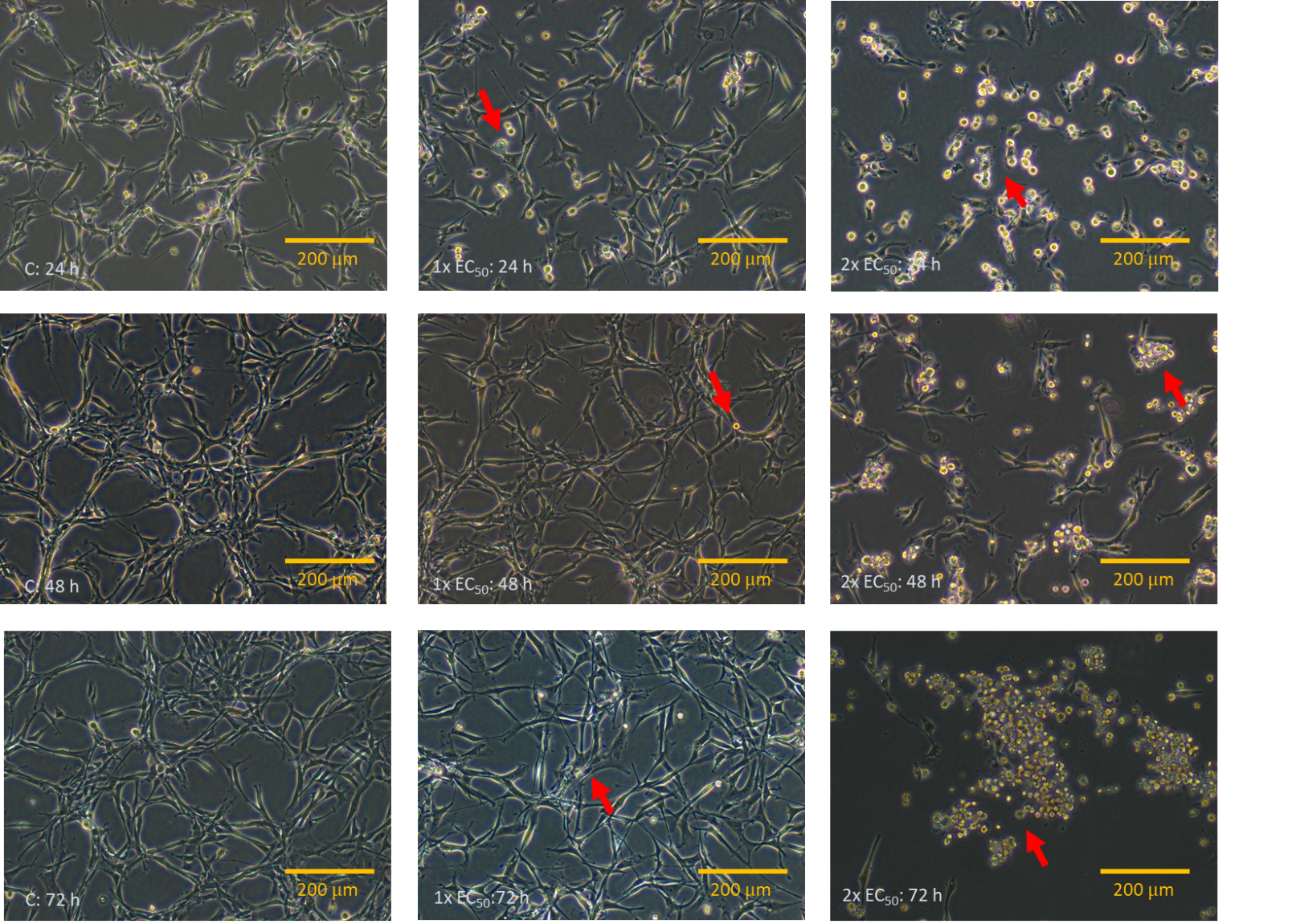


Supplementary Figure 2: U-87 MG cell morphology following MS13 treatment at concentration 1 $\times$ EC_50_ (7 $\mu$M) and 2 $\times$ EC_50_ (14 $\mu$M) compared with control (C) at treatment point of 24, 48 and 72 hours. The cell image was taken at 100× magnification. Red arrows indicate floating cells observed in MS13 treatment flask. These phase contrast cell images were captured using inverted microscope CKX14-A22 (Olympus, Japan).

Supplementary Table 1: List of differentially expressed proteins (DEPs) identified in U-87 MG cells treated with 14 µM of MS13 for 24 hours and their respective PANTHER protein class.

| **Protein name/ Protein class** | **UniProt ID** | **Gene/ Protein symbol** | **Significance** | **p-value** | **Group profile (Ratio)** | **Coverage (%)** | **# Peptide** | **# Unique peptide** | **Average mass** |
| --- | --- | --- | --- | --- | --- | --- | --- | --- | --- |
| **Up-regulated proteins** | | | | | | | | | |
| **Chaperone** |  |  |  |  |  |  |  |  |  |
| Heat shock protein HSP 90-alpha* | P07900 | *HSP90AA1*/ HSP90A | 24.87 | 3.26 $\times$ 10^-3^ | 1.00: 1.50 | 15 | 9 | 5 | 84660 |
| Heat shock protein HSP 90-beta* | P08238 | *HSP90AB1*/ HSP90B | 23.11 | 4.89 $\times$ 10^-3^ | 1.00: 1.48 | 10 | 6 | 2 | 83264 |
| Endoplasmic reticulum chaperone BiP | P11021 | *HSPA5*/ BIP | 21.09 | 7.78 $\times$ 10^-3^ | 1.00: 1.45 | 4 | 2 | 1 | 72333 |
| 14-3-3 protein theta | P27348 | *YWHAQ*/ 1433T | 23.58 | 4.39 $\times$ 10^-3^ | 1.00: 1.48 | 16 | 3 | 2 | 27764 |
| **Cytoskeletal binding protein** |  |  |  |  |  |  |  |  |  |
| Myosin regulatory light chain 12A | P19105 | *MYL12A*/ MYL12A | 30.25 | 9.44 $\times$ 10^-4^ | 1.00: 1.58 | 6 | 1 | 1 | 19794 |
| Myosin regulatory light chain 12B | O14950 | *MYL12B*/ MYL12B | 30.25 | 9.44 $\times$ 10^-4^ | 1.00: 1.58 | 6 | 1 | 1 | 19779 |
| Filamin-C | Q14315 | *FLNC*/ FLNC | 14.98 | 3.18 $\times$ 10^-2^ | 1.00: 1.35 | 1 | 3 | 1 | 291020 |

Supplementary Table 1 Continue 1

| **Protein name/ Protein class** | | **UniProt ID** | **Gene/ Protein symbol** | **Significance** | **p-value** | **Group profile (Ratio)** | | | | **Coverage (%)** | | | **# Peptide** | | **# Unique peptide** | | | | **Average mass** |
| --- | --- | --- | --- | --- | --- | --- | --- | --- | --- | --- | --- | --- | --- | --- | --- | --- | --- | --- | --- |
| **Up-regulated proteins (cont.)** | | | | | | | | | | | | | | | | | | | |
| **Histone** | |  |  |  |  |  | | | |  | | |  | |  | | | |  |
| Histone H2AX* | | P16104 | *H2AFX*/ H2AFX | 22.91 | 5.12 $\times$ 10^-3^ | 1.00: 1.47 | | | | 27 | | | 5 | | 2 | | | | 15145 |
| **Metabolic enzyme** | |  |  |  |  |  | | | |  | | |  | |  | | | |  |
| Malate dehydrogenase mitochondrial | | P40926 | *MDH2*/ MDHM | 33.94 | 4.04 $\times$ 10^-4^ | 1.00: 1.63 | | | | 9 | | | 2 | | 2 | | | | 35503 |
| **Down-regulated proteins** | | | | | | | | | | | | | | | | | | | |
| **RNA binding protein** | |  |  |  |  |  | |  | | |  | | |  | | |  | | |
| Heterogenous nuclear ribonucleoprotein K* | | P61978 | *HNRNPK*/ HNRNPK | 16.69 | 2.14 $\times$ 10^-2^ | 1.00: 0.73 | | | | 12 | | | 3 | | 3 | | | | 50976 |
| Elongation factor 2 | | P13639 | *EEF2*/ EF2 | 75.4 | 2.88 $\times$ 10^-8^ | 1.00: 0.46 | | | | 8 | | | 4 | | 4 | | | | 95338 |
| Eukaryotic translation initiation factor 5A-1-like | Q6IS14 | | *EIF5AL1*/ IF5AL | 48.05 | 1.57 $\times$ 10^-5^ | | 1.00: 0.55 | | 8 | | | 1 | | | | 1 | | 16773 | |
| Eukaryotic translation initiation factor 5A-1* | | P63241 | *EIF5A*/ IF5A1 | 48.05 | 1.57 $\times$ 10^-5^ | 1.00: 0.55 | | | | 8 | | | 1 | | 1 | | | | 16832 |

Supplementary Table 1 Continue 2

| **Protein name/ Protein class** | **UniProt ID** | **Gene/ Protein symbol** | **Significance** | **p-value** | **Group profile (Ratio)** | **Coverage (%)** | **# Peptide** | **# Unique peptide** | **Average mass** |
| --- | --- | --- | --- | --- | --- | --- | --- | --- | --- |
| **Down-regulated proteins (cont.)** | | | | | | | | | |
| **RNA binding protein (cont.)** |  |  |  |  |  |  |  |  |  |
| Eukaryotic translation initiation factor 5A-2 | Q9GZV4 | *EIF5A2*/ IF5A2 | 48.05 | 1.57 $\times$ 10^-5^ | 1.00: 0.55 | 8 | 1 | 1 | 16793 |
| **Metabolic enzyme** |  |  |  |  |  |  |  |  |  |
| Glyceraldehyde-3-phosphate dehydrogenase* | P04406 | *GAPDH*/ G3P | 13.11 | 4.89 × 10^-2^ | 1.00: 0.55 | 29 | 6 | 6 | 36053 |
| Triosephosphate isomerase | P60174 | *TPI1*/ TPIS | 28.48 | 1.42 × 10^-3^ | 1.00: 0.64 | 44 | 8 | 8 | 30791 |
| Alpha-enolase* | P06733 | *ENO1*/ ENOA | 21.76 | 6.67 × 10^-3^ | 1.00: 0.69 | 24 | 6 | 5 | 41769 |
| Peroxiredoxin-1 | Q06830 | *PRDX1*/ PRDX1 | 36.43 | 2.28 × 10^-4^ | 1.00: 0.60 | 9 | 2 | 2 | 22110 |
| **Chaperone** |  |  |  |  |  |  |  |  |  |
| Endoplasmin | P14625 | *HSP90B1*/ ENPL | 30.29 | 9.35 × 10^-4^ | 1.00: 0.63 | 1 | 1 | 1 | 92469 |

Supplementary Table 1 Continue 3

| **Protein name/ Protein class** | | **UniProt ID** | | | **Gene/ Protein symbol** | | **Significance** | **p-value** | | **Group profile (Ratio)** | | **Coverage (%)** | **# Peptide** | | **# Unique peptide** | | **Average mass** | |
| --- | --- | --- | --- | --- | --- | --- | --- | --- | --- | --- | --- | --- | --- | --- | --- | --- | --- | --- |
| **Down-regulated proteins (cont.)** | | | | | | | | | | | | | | | | | | |
| **Chaperone (cont.)** | | | | | | | | | | | | | | | | | | |
| 14-3-3 protein zeta/delta | | P63104 | | | *YWHAZ*/ 1433Z | | 16.19 | 2.40 × 10^-2^ | | 1.00: 0.73 | | 17 | 3 | | 2 | | 27745 | |
| **Histone** | |  | |  | | |  |  | |  | |  |  | |  | |  | |
| Histone H2B type 1-B | | P33778 | | *HIST2H2BE*/ H2B1B | | | 22.37 | 5.79 × 10^-3^ | | 1.00: 0.68 | | 26 | 3 | | 1 | | 13950 | |
| Histone H2B type 1-J | | P06899 | | *HIST1H2BJ*/ H2B1J | | | 22.37 | 5.79 × 10^-3^ | | 1.00: 0.68 | | 26 | 3 | | 1 | | 13904 | |
| **Cytoskeletal protein** | |  | |  | | |  |  | |  | |  |  | |  | |  | |
| Talin-1 | | Q9Y490 | | *TLN1*/ TLN1 | | | 44.29 | 3.72 × 10^-5^ | | 1.00: 0.56 | | 7 | 2 | | 2 | | 269765 | |
| Tubulin beta chain* | | P07437 | | *TUBB*/ TBB5 | | | 23.12 | 4.88 × 10^-3^ | | 1.00: 0.68 | | 21 | 6 | | 1 | | 49671 | |
| **Calcium-binding protein** |  | |  | | |  | | |  |  |  | | |  | |  | |  |
| Annexin A1 | P04083 | | *ANXA1*/ ANXA1 | | | 26.11 | | | 2.45 × 10^-3^ | 1.00: 0.66 | 23 | | | 5 | | 5 | | 38714 |

Supplementary Table 1 Continue 4

| **Protein name/ Protein class** | **UniProt ID** | **Gene/ Protein symbol** | **Significance** | **p-value** | **Group profile (Ratio)** | **Coverage (%)** | **# Peptide** | **# Unique peptide** | **Average mass** |
| --- | --- | --- | --- | --- | --- | --- | --- | --- | --- |
| **Down-regulated proteins (cont.)** | | | | | | | | | |
| **Ribosomal protein** |  |  |  |  |  |  |  |  |  |
| 40S acidic ribosomal protein S13 | P62277 | *RPS13*/ RS13 | 47.70 | 1.70 × 10^-5^ | 1.00: 0.55 | 10 | 1 | 1 | 17222 |
| **Enzyme modulator** |  |  |  |  |  |  |  |  |  |
| Protein SET* | Q01105 | *SET*/ SET | 18.28 | 1.49 × 10^-2^ | 1.00: 0.71 | 8 | 2 | 2 | 33489 |
| **Cytoskeletal binding protein** |  |  |  |  |  |  |  |  |  |
| Profilin-1 | P07737 | *PFN1*/ PROF1 | 20.27 | 9.40 × 10^-3^ | 1.00: 0.38 | 21 | 2 | 2 | 15054 |

*Common differentially expressed proteins (DEPs) identified in both U-87 MG and SH-SY5Y cell

Supplementary Table 2: List of differentially expressed proteins (DEPs) identified in SH-SY5Y cells treated with 10 µM of MS13 for 48 hours and their respective PANTHER protein class.

| **Protein class/ Protein name** | **UniProt ID** | **Gene/ Protein symbol** | **Significance** | **p-value** | **Group profile (Ratio)** | **Coverage (%)** | **# Peptide** | **# Unique peptide** | **Average mass** |
| --- | --- | --- | --- | --- | --- | --- | --- | --- | --- |
| **Up-regulated proteins** | | | | | | | | | |
| **Chaperone** |  |  |  |  |  |  |  |  |  |
| 10 kDa heat shock protein mitochondrial | P61604 | *HSPE1*/ CH10 | 35.32 | 2.93 × 10^-4^ | 1.00:1.42 | 42 | 4 | 4 | 10932 |
| 60 kDa heat shock protein mitochondrial | P10809 | *HSPD1*/ CH60 | 13.81 | 4.16 × 10^-2^ | 1.00:1.47 | 13 | 5 | 5 | 61055 |
| Heat shock protein beta-1 | P04792 | *HSPB1*/ HSPB1 | 39.13 | 1.22 × 10^-4^ | 1.00:1.70 | 9 | 2 | 2 | 22783 |
| Heat shock protein HSP 90-alpha* | P07900 | *HSP90AA1*/ HSP90A | 13.19 | 4.80 × 10^-2^ | 1.00:1.44 | 11 | 8 | 4 | 84660 |
| Heat shock protein HSP 90-beta* | P08238 | *HSP90AB1*/ HSP90B | 36.36 | 2.31 × 10^-4^ | 1.00:1.31 | 13 | 8 | 4 | 83264 |
| **Metabolic enzymes** |  |  |  |  |  |  |  |  |  |
| Isochorismatase domain-containing protein 1 | Q96CN7 | *ISOC1*/ ISOC1 | 26.98 | 2.00 × 10^-3^ | 1.00:1.53 | 5 | 1 | 1 | 32237 |

Supplementary Table 2 Continue 1

| **Protein class/ Protein name** | **UniProt ID** | **Gene/ Protein symbol** | **Significance** | **p-value** | **Group profile (Ratio)** | **Coverage (%)** | **# Peptide** | **# Unique peptide** | **Average mass** |
| --- | --- | --- | --- | --- | --- | --- | --- | --- | --- |
| **Up-regulated proteins (cont.)** | | | | | | | | | |
| **Cytoskeletal binding protein** |  |  |  |  |  |  |  |  |  |
| Myosin-9 | P35579 | *MYH9*/ MYH9 | 36.79 | 2.09 × 10^-4^ | 1.00:1.67 | 1 | 1 | 1 | 226530 |
| **Down-regulated proteins** | | | | | | | | | |
| **Histone** |  |  |  |  |  |  |  |  |  |
| Histone H1.5 | P16401 | *HIST1H1B*/ H15 | 19.9 | 1.02 × 10-2 | 1.00:0.34 | 6 | 1 | 1 | 22580 |
| Histone H2A type 1-A | Q96QV6 | *HIST1H2AA* / H2A1A | 40.72 | 8.47 × 10-5 | 1.00:0.58 | 30 | 5 | 3 | 14234 |
| Histone H2A.V | Q71UI9 | *H2AFV* / H2AV | 126.94 | 2.02 × 10^-13^ | 1.00:0.36 | 20 | 3 | 1 | 13509 |
| Histone H2AX* | P16104 | *H2AFX* / H2AX | 40.75 | 8.41 × 10^-5^ | 1.00:0.58 | 27 | 5 | 3 | 15145 |
| Histone H4 | P62805 | *HIST1H4A* / H4 | 84.99 | 3.17 × 10^-9^ | 1.00:0.44 | 41 | 4 | 4 | 11367 |

Supplementary Table 2 Continue 2.

| **Protein class/ Protein name** | **UniProt ID** | **Gene/ Protein symbol** | **Significance** | **p-value** | **Group profile (Ratio)** | **Coverage (%)** | **# Peptide** | **# Unique peptide** | **Average mass** |
| --- | --- | --- | --- | --- | --- | --- | --- | --- | --- |
| **Down-regulated proteins (Cont.)** | | | | | | | | | |
| **Cytoskeletal protein** |  |  |  |  |  |  |  |  |  |
| Actin cytoplasmic 1 | P60709 | *ACTB* / ACTB | 14.70 | 3.39 × 10^-2^ | 1.00:0.75 | 34 | 9 | 5 | 41737 |
| Actin cytoplasmic 2 | P63261 | *ACTG1* / ACTG | 14.70 | 3.39 × 10^-2^ | 1.00:0.75 | 34 | 9 | 5 | 41793 |
| Tubulin beta chain* | P07437 | *TUBB* / TBB5 | 24.88 | 3.25 × 10^-3^ | 1.00:0.66 | 30 | 9 | 1 | 49671 |
| Tubulin beta-2B chain | Q9BVA1 | *TUBB2B* / TBB2B | 13.73 | 4.24 × 10^-2^ | 1.00:0.43 | 40 | 12 | 3 | 49953 |
| Vimentin | P08670 | *VIM* / VIME | 13.16 | 4.83 × 10^-2^ | 1.00:0.60 | 32 | 12 | 12 | 53652 |
| Prelamin-A/C | P02545 | *LMNA* / LMNA | 61.63 | 6.87 × 10^-7^ | 1.00:0.50 | 1 | 1 | 1 | 74140 |
| **Metabolic enzyme** |  |  |  |  |  |  |  |  |  |
| Glyceraldehyde-3-phosphate* dehydrogenase | P04406 | *GAPDH* / G3P | 25.66 | 2.72 × 10^-3^ | 1.00:0.66 | 24 | 5 | 5 | 36053 |
| Phosphoglycerate kinase 1 | P00558 | *PGK1* / PGK1 | 17.99 | 1.59 × 10^-2^ | 1.00:0.72 | 16 | 4 | 4 | 44615 |

Supplementary Table 2 Continue 3.

| **Protein class/ Protein name** | **UniProt ID** | **Gene/ Protein symbol** | **Significance** | **p-value** | **Group profile (Ratio)** | **Coverage (%)** | **# Peptide** | **# Unique peptide** | **Average mass** |
| --- | --- | --- | --- | --- | --- | --- | --- | --- | --- |
| **Down-regulated proteins (Cont.)** | | | | | | | | | |
| Alpha-enolase* | P06733 | *ENO1* / ENOA | 17.22 | 1.90 × 10^-2^ | 1.00:0.72 | 23 | 6 | 3 | 47169 |
| Peroxiredoxin-6 | P30041 | *PRDX6* / PRDX6 | 74.80 | 3.31 × 10^-8^ | 1.00:0.46 | 9 | 2 | 2 | 25035 |
| Protein disulfide-isomerase | P07237 | *P4HB* / PDIA1 | 13.35 | 4.62 × 10^-2^ | 1.00:0.76 | 2 | 1 | 1 | 57116 |
| RNA binding protein |  |  |  |  |  |  |  |  |  |
| Heterogenous nuclear ribonucleoprotein K* | P61978 | *HNRNPK* / HNRNPK | 22.94 | 5.08 × 10^-3^ | 1.00:0.68 | 19 | 5 | 5 | 50976 |
| Heterogenous nuclear ribonucleoprotein U | Q00839 | *HNRNPU* / HNRNPU | 31.56 | 6.98 × 10^-4^ | 1.00:0.62 | 3 | 2 | 2 | 90585 |
| Heterogenous nuclear ribonucleoproteins C1/C2 | P07910 | *HNRNPC* / HNRNPC | 13.25 | 4.73 × 10^-2^ | 1.00:0.76 | 8 | 2 | 2 | 33670 |
| Eukaryotic translation initiation factor 5A-1* | P63241 | *EIF5A* / IF5A1 | 22.39 | 5.77 × 10^-3^ | 1.00:0.75 | 8 | 1 | 1 | 16832 |

Supplementary Table 2 Continue 4.

| **Protein class/ Protein name** | **UniProt ID** | **Gene/ Protein symbol** | **Significance** | **p-value** | **Group profile (Ratio)** | **Coverage (%)** | **# Peptide** | **# Unique peptide** | **Average mass** |
| --- | --- | --- | --- | --- | --- | --- | --- | --- | --- |
| **Down-regulated proteins (cont.)** | | | | | | | | | |
| **Calcium-binding protein** |  |  |  |  |  |  |  |  |  |
| Calmodulin-1 | P0DP23 | *CALM1* / CALM1 | 25.63 | 2.74 × 10^-3^ | 1.00:0.66 | 11 | 2 | 2 | 16838 |
| Calmodulin-2 | P0DP24 | *CALM2* / CALM2 | 25.63 | 2.74 × 10^-3^ | 1.00:0.66 | 11 | 2 | 2 | 16838 |
| Calmodulin-3 | P0DP25 | *CALM3* / CALM3 | 25.63 | 2.74 × 10^-3^ | 1.00:0.66 | 11 | 2 | 2 | 16838 |
| **Enzyme modulator** |  |  |  |  |  |  |  |  |  |
| Protein SET* | Q01105 | *SET* / SET | 107.93 | 1.61 × 10^-11^ | 1.00:0.37 | 3 | 1 | 1 | 33489 |
| Cell division control protein 42 homolog | P60953 | *CDC42* / CDC42 | 13.11 | 4.89 × 10^-2^ | 1.00:0.76 | 7 | 1 | 1 | 21259 |
| **Ribosomal protein** |  |  |  |  |  |  |  |  |  |
| 60S acidic ribosomal protein P2 | P05387 | *RPLP2* / RLA2 | 33.31 | 4.67 × 10^-4^ | 1.00:0.62 | 56 | 3 | 3 | 11665 |

Supplementary Table 2 Continue 5.

| **Protein class/ Protein name** | **UniProt ID** | **Gene/ Protein symbol** | **Significance** | **p-value** | **Group profile (Ratio)** | **Coverage (%)** | **# Peptide** | **# Unique peptide** | **Average mass** |
| --- | --- | --- | --- | --- | --- | --- | --- | --- | --- |
| **Down-regulated proteins (Cont.)** | | | | | | | | | |
| **Cytoskeletal binding protein** |  |  |  |  |  |  |  |  |  |
| Stathmin | P16949 | *STMN1* / STMN1 | 115.09 | 3.10 × 10^-12^ | 1.00:0.38 | 15 | 2 | 2 | 17302 |
| **Cell adhesion molecule** |  |  |  |  |  |  |  |  |  |
| Galectin-1 | P09382 | *LGALS1* / LEG1 | 32.13 | 6.12 × 10^-4^ | 1.00:0.62 | 12 | 2 | 2 | 14716 |
| **Cation transporter** |  |  |  |  |  |  |  |  |  |
| ATP synthase subunit beta mitochondrial | P06576 | *ATP5F1B* / ATPB | 58.80 | 1.32 × 10^-6^ | 1.00:0.51 | 5 | 2 | 2 | 56560 |
| **Chaperone** |  |  |  |  |  |  |  |  |  |
| 1433E_HUMAN | P62258 | *YWHAE* / 1433E | 19.13 | 1.22 × 10^-2^ | 1.00:0.61 | 16 | 3 | 2 | 29174 |

*Common differentially expressed proteins (DEPs) identified in both U-87 MG and SH-SY5Y cell
